# Care-seeking behaviour of adolescents with knee pain: a population-based study among 504 adolescents

**DOI:** 10.1186/1471-2474-14-225

**Published:** 2013-07-30

**Authors:** Michael S Rathleff, Sune K Skuldbøl, Mads N B Rasch, Ewa M Roos, Sten Rasmussen, Jens L Olesen

**Affiliations:** 1 Health, Aarhus University, Aarhus, Denmark; 2 Orthopaedic Surgery Research Unit, Aalborg University Hospital, Aalborg, Denmark; 3 Department of Rheumatology, Aalborg University Hospital, Aalborg, Denmark; 4 Institute of Sports Science and Clinical Biomechanics, University of Southern Denmark, Odense, Denmark

**Keywords:** Adolescents, Knee pain, Care-seeking, Treatment

## Abstract

**Background:**

Knee pain is common during adolescence. Adolescents and their parents may think that knee pain is benign and self-limiting and therefore avoid seeking medical care. However, long-term prognosis of knee pain is not favourable and treatment seems to offer greater reductions in pain compared to a “wait-and-see” approach. The purpose of this study was to describe the determinants of care-seeking behaviour among adolescents with current knee pain and investigate what types of treatment are initiated.

**Methods:**

An online questionnaire was forwarded to 2,846 adolescents aged 15–19 in four upper secondary schools. The questionnaire contained questions on age, gender, height, weight, currently painful body regions, frequency of knee pain, health-related quality of life measured by the EuroQol 5-dimensions, sports participation and if they had sought medical care. Adolescents who reported current knee pain at least monthly or more frequently were telephoned. The adolescents were asked about pain duration, onset of knee pain (traumatic or insidious) and if they were currently being treated for their knee pain.

**Results:**

504 adolescents currently reported at least monthly knee pain. 59% of these had sought medical care and 18% were currently under medical treatment . A longer pain duration and higher pain severity increased the odds of seeking medical care. Females with traumatic onset of knee pain were more likely to have sought medical care than females with insidious onset of knee pain. Females with traumatic onset of knee pain and increased pain severity were more likely to be undergoing medical treatment. The most frequently reported treatments were the combination of exercises and orthotics (68% of those undergoing medical treatment).

**Conclusion:**

Females with insidious onset of knee pain do not seek medical care as often as those with traumatic onset and adolescents of both genders with insidious onset are less likely to be under medical treatment. These findings are important as knee pain with insidious onset has similar consequences as knee pain with traumatic onset regarding pain severity, pain duration and reductions in health-related quality of life.

## Background

Epidemiological studies show that adolescent self-reported pain is frequent [1]. One of the most prevalent regions of pain is the knee [2-4]. Between 19 and 31% of adolescents report knee pain [3,5,6] with Patellofemoral Pain (PFP) being one of the most common knee conditions among adolescents with a prevalence of approximately 7% [6-8]. Adolescents may develop knee pain after a sudden unexpected traumatic injury or with an insidious slowly developing onset where the adolescent or parent does not know what initiated the pain [9]. An insidious onset of knee pain among adolescents is usually regarded as self-limiting and therefore intervention has been questioned [10-12].

Adolescent knee pain does not always have a favourable long-term prognosis. Nimon et al. [13] followed a group of females with insidious onset of anterior knee pain and found that 78% still reported knee pain after 14–20 years [13]. Longer pain duration before initiation of treatment is associated with poorer long-term prognosis among patients with PFP [14] and treatment by foot orthotics results in greater reductions in pain compared to a “wait-and-see” approach [15]. As the long-term prognosis of knee pain is not favourable and treatment seems to offer greater reductions in pain compared to a “wait-and-see” approach [15], it is important to investigate the care-seeking behaviour among adolescents with knee pain and what types of treatment are initiated.

The purpose of this study was therefore to describe the determinants of care-seeking behaviour among adolescents with current knee pain and to investigate how many adolescents with current knee pain are currently under medical treatment for their knee pain.

## Methods

### Design

Adolescents were recruited from a population-based cohort (Adolescent Pain in Aalborg 2011, the APA2011-cohort), which consists of 4,007 adolescents aged 12–19 years. In this analysis, only adolescents from the upper secondary schools were included (2,846 adolescents, 71% of the entire cohort) [16]. Two papers have previously been published from the APA2011-cohort. They describe pain and muscular mechanisms in the subsample diagnosed with PFP [8,17]. Ethical approval was obtained from the local ethics committee in the North Denmark Region (N-20110020). The ethics committee did not require an individual signed consent, but required that the schools informed the parents about the study and that participation in the study was voluntary. The reporting of the study follows the “Strengthening the Reporting of Observational studies in Epidemiology” (STROBE statement) [18].

### Study population

There are four upper secondary schools in the area where the study was conducted. The four schools all have students from low, middle and high socioeconomic status. However no individual specific socioeconomic status was obtained. All four schools agreed to participate and all adolescents were invited to answer an online questionnaire as part of their physical education lessons. Adolescents currently exempted from physical education because of pain or similar conditions still participated in the study.

### Procedure

Before data collection, the primary author [MSR] visited all schools that agreed to participate and told them about the purpose of the study and instructed them regarding the content of the questionnaire. A leaflet was distributed to adolescents with the title “*Please help answer a questionnaire on a scientific study on physical activity, quality of life and pain*”. The leaflet contained information that the study was done by the Orthopaedic Surgery Research Unit at the University Hospital together with the Graduate School of Health Sciences at Aarhus University. In addition, a detailed description inside the leaflet contained information on the interest of the association between physical activity, quality of life and musculoskeletal pain, but especially knee pain.

The online questionnaire contained demographic questions on age, gender, height, weight, which school they attended, if they participated in sports in their leisure time and health related quality of life measured by the EuroQol 5-dimensions (EQ-5D) [19]. After answering these questions, the adolescents were presented with a pain mannequin. The pain mannequin was shown with a frontal and posterior view showing the front and the back of a human body divided into 12 predefined regions [20]. For further details on the online questionnaire and recruitment please refer to Rathleff et al. [16]. The adolescents were instructed to mark the regions where they experienced current pain [20] and report the frequency of pain (divided into: rarely, monthly, weekly, more than one time per week, almost daily pain) [21]. They were also asked if they had consulted their general practitioner (GP) regarding pain in that specific region.

### Adolescents with self-reported knee pain

Adolescents who reported knee pain at least monthly, were telephoned by a physiotherapist. Adolescents who did not respond to our telephone call were called an additional two times. If the adolescents did not return our call, a text message was sent to their mobile telephone explaining that we would like to ask them a few additional questions regarding the online questionnaire. If the adolescents did not respond to the text message, they were called once more.

The physiotherapist asked the adolescent about 1) the time of onset of their current knee pain, 2) if the knee pain started after trauma or it had an insidious onset, and 3) if they were currently receiving treatment for their knee pain. If they were currently receiving treatment, they were asked by whom and which type of treatment they were receiving. All responses were transcribed as closely as possible to the adolescents` own wording to avoid interpretation bias.

### Interpretation of the adolescents’ response

Adolescents who reported knee pain with a duration of “a couple of years” was interpreted as 24 months. If they responded they had had pain for “as long as I can remember” or “always” it was interpreted as 120 months. If the adolescent could not remember how their knee pain started, the physiotherapist asked if they could remember a specific event where they first felt their knee pain. If the adolescent said no, they were asked if the knee pain slowly developed without a clear onset. If they did not know if their knee pain was related to a traumatic or insidious onset, it was interpreted as “insidious”. A total of 11 adolescents could not remember if their knee pain was initiated by a traumatic event or it had an insidious onset and were therefore interpreted as “insidious onset”.

All the following answers were interpreted as “currently under treatment”: if the adolescents had received an exercise program by a physiotherapist or general practitioner (GP) and still performed the exercises; surgery and postoperative exercise; foot or knee orthotics prescribed by GP or physiotherapist; if the GP had prescribed NSAID. The following was interpreted as “not currently under treatment”: if the adolescents reported they were referred for investigations at the hospital, by a GP or by a physiotherapist; if the adolescent reported they had consulted their GP and received advice to decrease physical activity.

### Data analysis

Demographics, participation in sports, pain severity, pain duration, percentage who sought medical care, percentage who were under treatment and EQ-5D score were stratified for gender and onset of knee pain. Adolescents with traumatic onset of knee pain were compared with adolescents with insidious onset of knee pain using Student’s t-test, Wilcoxon Rank Sum test or proportion test depending on data type. The association between “seeking medical care” and “currently under treatment”, respectively, and the dependant variables: gender, EQ-5D (categorised in quartiles based on EQ-5D index score), body mass index (BMI; categorised into quartiles), pain duration, onset of pain (traumatic versus insidious onset) were tested through logistic regression analyses using robust variance estimates that adjust for within-cluster correlations within schools [22]. All 504 adolescents were included in both logistic regression analyses. Categorical dependent variables were entered into the logistic regression analyses by using dichotomous indicator variables using the built-in indicator function of Stata.

Furthermore “Contact to GP” was included in the model with the independent variable “currently under treatment”. Model construction was done according to Hosmer and Lemeshow’s “Purposeful selection of variables” [23]. Interaction between gender and all dependent variables were tested. Interaction between onset of knee pain and gender was found in the analysis with “seeking medical care” as the independent variable. P < 0.05 was considered statistically significant and no adjustments were made for multiple comparisons [24]. Stata (Version 11) was used for all statistical analyses.

## Results

### Non-responder analysis

**Figure 1:**
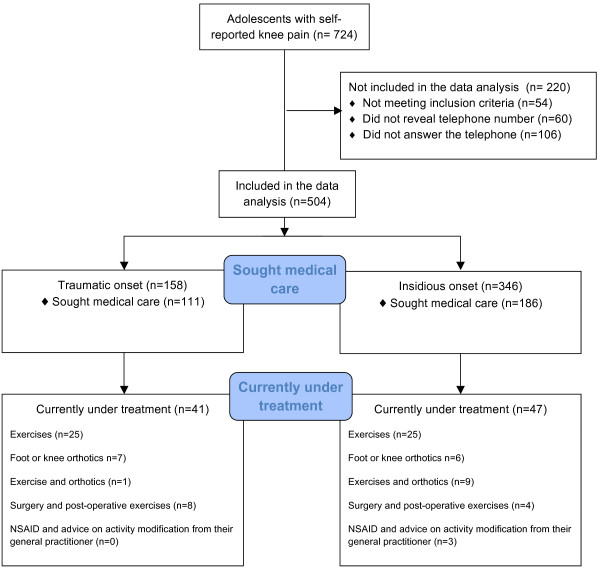
Flow-chart shows adolescents with an insidious or a traumatic onset of knee pain and the proportions that sought medical care and those who were currently being treated for their knee pain.

From 2,846 potential responders, 2,200 adolescents answered the questionnaire corresponding to a response rate of 77%. A total of 670 adolescents reported knee pain, at least monthly or more frequently. 60 adolescents did not report their telephone number in the questionnaire, leaving 610 potential responders. A total of 504 adolescents were successfully contacted by telephone (response rate of 83% of those who reported their telephone number; see Figure 1). A non-responder analysis using data from the online questionnaire was conducted. Responders (n = 504) had a higher pain severity with 28% reporting daily pain versus only 15% among non-responders (the other categories were: more than once per week 19% vs. 15%, weekly 29% vs. 22%, monthly 24% vs. 47% (p < 0.001). No difference was observed in gender distribution (70.5 vs. 73%, p = 0.583), median BMI (21.58 vs. 21.38, p = 0.934) or average EQ-5D index score (0.75 vs. 0.78, p = 0.093) compared to non-responders (n = 166).

### Demographics

The majority of the sample was female with a median age of 17 (Table 1). A total of 158 (31.7%) reported knee pain with traumatic onset while 346 (68.3%) reported insidious onset of pain (Table 2). An unadjusted analysis showed no difference in pain frequency between those with traumatic onset of pain and those with insidious onset of pain (p = 0.589).

**Table 1:** Demographics showing age, height, weight and Body Mass Index (BMI)*

	**Females, n = 363**	**Males, n = 141**
Age [years], median and (IQR)	17 (16–18)	17 (17–18)
Height [cm], mean (SD)	168.1 (±6.6)	182.4 (±6.4)
Weight [kg], mean (SD)	62.1 (±10.5)	73.5 (±9.8)
BMI [kg/m^2^], median and (IQR)	21.8 (20.2-23.8)	21.5 (19.6-23.5)

*Not cluster-adjusted.

**Table 2:** Demographics and key variables stratified into gender and onset of knee pain*

	**Traumatic onset**		**Insidious onset**		**Comparison between females**	**Comparison between males**
	**Female (n = 110)**	**Male (n = 50)**	**Female (n = 253)**	**Male (n = 91)**		
Age [years] median and (IQR)	17 (17–18)	18 (17–18)	17 (16–18)	17 (17–18)	0.138	0.132
Height [cm], mean (95%CI)	168.2 (166.9-169.5)	182.7 (181.1-184.9)	168.1 (167.2-168.9)	182.3 (181.0-183.6)	0.903	0.720
Weight [kg], mean (95%CI )	64.2 (62.1-66.3)	74.7 (71.8-77.6)	61.1 (59.9-62.4)	72.6 (70.6-74.5)	0.012	0.211
BMI [kg/m^2^], median (QR)	22.1 (20.2-24.8)	22.2 (20.7-24.5)	21.1 (19.5.2-23.1)	21.6 (20.2-23.5)	0.003	0.232
Gender distribution [% of all in the cohort] (95%CI)	21.8 (14.1-29.5)	9.9 (1.6-18.2)	50.2 (44.0-56.4)	18.1 (10.2-26.0)	<0.0001	0.19
Participation in sports during leisure time [% who answered yes] (95%CI)	67.0 (58.1-75.9)	75.5 (63.3-87.7)	70.7 (65.0-76.4)	76.7 (67.9-85.5)	0.478	0.878
Pain frequency (%)						
Daily	36.4	16.0	25.3	26.4	0.067	0.201
More than once per week	17.3	14.0	23.3	13.1		
Weekly	30.0	30.0	26.9	36.3		
Monthly	16.4	41.0	24.5	24.2		
Pain duration [months], median (IQR)	24 (12–42)	18 (10–36)	24 (12–48)	24 (12–42)	0.382	0.241
Have you sought medical care? [% who replied yes] (95%CI)	80.0 (72.5-87.5)	47.9 (33.6-62.2)	55.7 (49.6-61.9)	49.5 (39.1-59.8)	<0.0001	0.863
Currently under treatment? [% who replied yes] (95%CI)	28.2 (19.7-36.6)	20.0 (8.8-31.2)	13.4 (9.2-17.7)	14.3 (7.0-21.5)	0.0001	0.524
EQ-5D index score, median (IQR)	0.78 (0.71-0.82)	0.78 (0.76-0.82)	0.78 (0.72-0.82)	0.78 (0.78-0.82)	0.246	0.8984
EQ-5D-vas, median (IQR)	72 (60–82)	75 (65–88)	72 (54–85)	79 (68–88)	0.868	0.619

*Not cluster adjusted.

### Seeking medical care

59% of the adolescents sought medical care for their knee pain. The unadjusted analysis showed that EQ-5D, pain severity, onset of knee pain and pain duration were associated with seeking medical care. The adjusted analysis showed that increased pain severity, longer pain duration and lower EQ5D index score increased the odds of having sought medical care (Table 3). There was a significant interaction between onset of pain and gender indicating females who had traumatic onset of knee pain were more likely to seek medical care than females with insidious onset of knee pain. This effect was not seen among males indicating that males with insidious onset of knee pain were as likely as those with traumatic onset of pain to seek medical care.

**Table 3:** Odds for having sought medical care

	**Crude**	**p-value**	**Adjusted**	**p-value**	**[95% CI]**
	**OR**	**OR**	**OR**	**Adj OR**	**Adj OR**
Older age (15 years of as referent)	1.16	0.176	1.09	0.531	0.83-1.43
Female gender	1.79	0.056	1.21	0.564	0.63-2.34
Participation in sports (compared to not participating in sports)	0.76	0.182	1.01	0.957	0.70-1.45
BMI (compared to 0-25% quartile, 16.18; 19.71)					
2. quartile (19.71; 21.55)	1.03	0.881	0.93	0.824	0.48-1.78
3. quartile (>21.55; 23.62)	1.30	0.030	1.12	0.597	0.73-1.72
4. quartile (>23.62; 34.00)	1.53	0.083	1.20	0.431	0.76-1.91
EQ-5D index score (compared to 0-25% quartile, -0.169;0.723)					
25-50% percentile (>0.723; 0.776)	0.64	0.066	0.93	0.765	0.57-1.50
50-75% percentile (>0.776; 0.824)	0.34	<0.001	0.66	0.013	0.48-0.92
75-100% percentile (>0.824; 1.000)	0.35	0.001	0.68	0.015	0.50-0.93
Pain severity (compared to daily pain)					
More than once per week	0.41	<0.001	0.45	<0.001	0.29-0.69
Weekly	0.31	<0.001	0.40	<0.001	0.32-0.50
Monthly	0.15	<0.001	0.19	<0.001	0.11-0.32
Pain duration (per 10 month increase)	1.15	<0.001	1.15	<0.001	1.08-1.23
Traumatic onset of pain (compared to an insidious onset)	1.98	0.001			
Traumatic onset of knee pain compared to insidious onset among females			2.75	0.031	1.10-6.91
Traumatic onset of knee pain compared to insidious onset among males			1.15	0.738	0.51-2.61

Univariate and multivariate logistic regression analysis. Crude OR shows the result from the univariate logistic regression analysis between “having sought medical care” and each of the explanatory variables. Adjusted coefficients show the results from the multivariate analysis.

### Treatment

47 adolescents with an insidious onset of knee pain were currently under treatment with 38 (81%) of those receiving exercise, with or without the addition of surgery. An additional 6 (13%) adolescents were referred for further investigation at the hospital and 3 (13%) received advice from their GP on reducing their activity level. Among adolescents with traumatic onset of knee pain, 41 were currently under treatment with 34 (83%) receiving exercise, with or without the addition of surgery. An additional 8 (20%) adolescents were referred for further investigation at the hospital (Figure 1).

The unadjusted analysis showed that EQ-5D, pain severity, onset of knee pain and contact to GP were associated with the odds of being under medical treatment. The adjusted analysis showed the 2nd and 3rd quartile BMI were more likely to be under treatment compared to the lowest quartile (Table 4). Adolescents with monthly knee pain were less likely to be under medical treatment compared with those with daily knee pain (Table 4). Reporting traumatic onset of knee pain was associated with increased odds of being under medical treatment.

**Table 4:** Odds for currently being under medical treatment

	**Crude**	**p-value**	**Adjusted**	**p-value**	**[95% CI]**
	**OR**	**OR**	**OR**	**Adj OR**	**Adj OR**
Older age (15 years of as referent)	1.13	0.074	1.06	0.431	0.91-1.24
Female gender	1.26	0.068	0.97	0.871	0.70-1.34
Participation in sports (compared to not participating in sports)	0.66	0.320	0.77	0.529	0.34-1.73
BMI (compared to 0-25% quartile, 16.18; 19.71)					
2. quartile (19.71; 21.55)	1.64	0.015	1.75	<0.001	1.30-2.35
3. quartile (>21.55; 23.62)	1.81	0.010	1.56	0.016	1.08-2.25
4. quartile (>23.62; 34)	1.50	0.188	1.16	0.666	0.57-2.35
EQ-5D index score (compared to 0–25 quartile, -0.169; 0.723)					
25-50% quartile (>0.723; 0.776)	0.69	0.051	0.94	0.801	0.60- 1.48
50-75 quartile (>0.776; 0.824)	0.40	<0.001	0.76	0.201	0.51-1.15
75-100% quartile (>0.824; 1.000)	0.77	0.338	1.47	0.067	0.97-2.21
Pain severity (compared to daily pain)					
More than once per week	0.48	<0.001	0.59	<0.001	0.45-0.75
Weekly	0.40	<0.001	0.53	<0.001	0.38-0.76
Monthly	0.17	<0.001	0.25	0.002	0.11-0.59
Pain duration (per 10 month increase)	1.05	0.131	1.01	0.737	0.95-1.08
Traumatic onset of pain (compared to an insidious onset)	2.27	0.032	2.24	0.034	1.06-4.72
Contact to GP	5.04	<0.001	3.64	0.002	1.64- 8.08

Univariate and multivariate logistic regression analysis. Crude OR shows the result from the univariate logistic regression analysis between “being under medical treatment” and each of the explanatory variables. Adjusted coefficients show the results from the multivariate analysis.

## Discussion

The main findings are that lower EQ-5D index score, higher pain severity and traumatic onset of knee pain were associated with both seeking medical care and being under treatment. Females with insidious onset of knee pain do not seek medical care as often as females with traumatic onset and adolescents of both genders with insidious onset are less likely to be under medical treatment. These findings are important as knee pain with insidious onset has similar consequences as traumatic onset regarding pain severity, pain duration and reductions in health-related quality of life. This is the first study that investigates knee pain among adolescents recruited from a large population-based cohort. This method of recruitment offers a unique possibility to study adolescent knee pain without the potential selection bias occurring as a result of recruitment through GP or specialised sports medicine clinics.

### Care-seeking behaviour

Almost 60% of the adolescents with knee pain had sought medical care. Care-seeking for knee pain seems similar to care-seeking for generalised pain, as the prevalence rates from this study are similar to those reported in previous studies of unspecific musculoskeletal pain. Roth-Isiqkeit et al. found that 51% of children and adolescents with pain had consulted their physician [25] while Perquin et al. [26] found that 57% had contacted their GP [26]. A lower prevalence was found in a Finnish study where only 16% of males and 20% of females aged 16 had sought medical care [27]. This discrepancy may be attributed to differences in health care systems and the nature of the pain being studied [27].

Previous studies have reported similar determinants of seeking medical care among adolescents. These determinants were high frequency of pain, female gender, high BMI, and lower self-rated health [26]. However no one has previously investigated how the onset of pain is associated with care-seeking behaviour. We found increased odds of seeking medical care after knee pain with traumatic onset. This is a novel finding, however it resembles the findings by De Inocencio [28] who reported that physical trauma was the most common aetiology of pain among adolescents visiting a primary care physician for musculoskeletal pain.

It has been speculated that adolescents reporting pain after a trauma are more likely to be physically active and participate in organised sports [27]. Those participating in organised sports may be more aware of a cause or medical diagnosis for their pain condition and have better access to medical care because of their sports insurance. Sports insurance is required in some countries to participate in organised sports, but not in Denmark. Our results question this finding as we did not find an association between sports participation during leisure time and the odds of seeking medical care. Another more obvious explanation is that adolescents are more concerned about knee pain as a result of a traumatic event compared to insidious pain that slowly develops over time without a clear starting point.

### Treatment

To our knowledge, no one has yet investigated which types of treatment are most frequently used to treat adolescents with knee pain in primary and secondary care. A study from Italy found that 96.8% of adolescents with non-specific musculoskeletal pain were advised to rest and use medication, seek physical therapy and rehabilitation, or a combination of these [29]. However they also found that only 70% of the adolescents complied with the advice. This may suggest that some of the adolescents from the current study sought medical care from their GP and were advised to visit a physiotherapist or reduce activity. However, the adolescents may not have followed the prescription or simply did not consider the “advice” a valid treatment. This may lead to an underreporting of the proportion currently being treated for their knee pain.

Another study investigated medical consumption for chronic non-specific pain among adolescents [26]. They found that pain intensity increased the usage of medication for treating adolescent pain. We also found that increased pain severity increased the odds of currently being treated for knee pain. In addition, we observed that a traumatic onset of knee pain increased the odds of currently being under medical treatment, even after adjusting for pain severity, EQ-5D and contact with their GP. This might be attributed to factors related to the adolescent, the GP or the severity of the injury.

### Strengths and weaknesses of the study

All data were self-reported and we did not obtain information from either parents or general practitioners. We used simple questions using a standardised electronic questionnaire protocol, but there could be some adolescents who misunderstood our questions. This can lead to misclassification bias. The cross-sectional design of the study increases the risk of recall bias and thereby the validity of our data on care-seeking behaviour, pain duration and onset of knee pain. The median pain duration was 24 months. Therefore, contacting the GP could have taken place a long time ago and the adolescents could have forgotten it. The same might hold true for the onset of knee pain. However, we do not believe that the information obtained on current treatment is influenced by such a bias, as this information is related to the present and involves no recall. No information was collected on which specific GP the adolescent was listed with. There may be a wide variation in GPs’ diagnosis and prescribed treatments and future studies should control for the potential heterogeneity between GPs.

One of the strengths of the study is that Danish citizens have free and unlimited access to health care through the GP. Therefore the care-seeking behaviour is not biased due to unequal access to health care. However, even though Denmark offers equal free access to heath care through the GP, we cannot rule out that socioeconomic state may influence our results. Although the equal access improves the external validity of the results to countries with similar health care systems, our findings may not be generalisable to countries with unequal access to health care.

### Implications for clinicians and researchers

General practitioners, adolescents and parents need to be aware that knee pain with insidious onset may be equally serious as knee pain with traumatic onset regarding pain severity, pain duration and reductions in health-related quality of life. Longer pain duration seems to be associated with a poorer long-term prognosis after initiation of treatment [14,30]. This seems to hold true for many different musculoskeletal disorders, including knee pain, and health personnel involved in treating adolescents with knee pain should consider intervening at an early stage of symptom onset From a research perspective, our findings are important as they suggest that recruitment of adolescents with knee pain should be done through population-based cohorts to avoid selection bias. The data from our study show that not all adolescents with knee pain will contact their GP. 40% of the true population would not be included if recruitment was done through the GP. The population-based recruitment used in the current study will enable future studies with high external validity.

### Future research

A large percentage of the adolescents had sought medical care, but only 18% were currently being treated, even though they all reported current knee pain. Future studies should aim to prospectively describe the clinical pathway of adolescents with knee pain in primary care and survey the number of consultations, the types of prescribed treatment and compliance. Further, we need to describe the reasons for treatment termination in primary and secondary care and investigate if it is related to an absence of pain or because no further reductions in pain can be achieved by intervening.

## Conclusion

Females with insidious onset of knee pain do not seek medical care as often as those with traumatic onset and adolescents of both genders with insidious onset are less likely to be under medical treatment. These findings are important as knee pain with insidious onset has similar consequences as knee pain with traumatic onset regarding pain severity, pain duration and reductions in health-related quality of life.

## Competing interests

The authors declare that they have no competing interests.

## Authors’ contributions

MSR planned the study together with EMR, SR and JLO. MSR, SKK, MNBR and JLO helped collect the data. MSR made the first draft of the result section while EMR, SR, JLO, SKK and MNBR provided important feedback on the interpretation of the results. MSR wrote the first draft of the manuscript while EMR, SR, JLO, SKK and MNBR provided feedback on the draft. All authors read and approved the final manuscript.

## Pre-publication history

The pre-publication history for this paper can be accessed here:

http://www.biomedcentral.com/1471-2474/14/225/prepub
